# An unusual case of disseminated cryptococcosis in an immunocompetent host presenting with verrucous skin lesions

**DOI:** 10.1016/j.mmcr.2023.100606

**Published:** 2023-08-29

**Authors:** Heloi Stefani, Laís Lopes Almeida Gomes, Fernanda Gonçalves Moya

**Affiliations:** a Federal University of São Paulo, Rua Sena Madureira, n.° 1.500 - Vila Clementino, São Paulo, SP, 04021-001, Brazil; b Massachusetts General Hospital, 55 Fruit St, Boston, MA, 02114, United States; c Hospital das Clínicas da Faculdade de Medicina da Universidade de São Paulo, R. Dr. Ovídio Pires de Campos, 225, Cerqueira César, São Paulo, SP, 05403-010, Brazil

**Keywords:** Disseminated cryptococcosis, Immunocompetent host, Verrucous lesion, *Cryptococcus gattii*, PET scan

## Abstract

Disseminated cryptococcosis, commonly linked to immunocompromised conditions like HIV infection, is exceedingly rare in immunocompetent individuals. This case report presents a rare case of disseminated cryptococcosis in an immunocompetent patient, who manifested with fever, weight loss, neurological manifestations, and distinct verrucous skin lesions. Mycological cultures and histopathological assessments were conducted, leading to the identification of *Cryptococcus neoformans* var. *gattii* within both lung and skin biopsies. This case highlights the significance of considering this yeast infection within immunocompetent individuals and the necessity for promptly initiating appropriate antifungal therapy to enhance patient outcomes.

## 1 Introduction

Cryptococcosis is an infection caused by encapsulated basidiomycetous yeasts, recognized as two distinct species: *C. neoformans,* and *C. gattii.* Environmental exposures described regarding interactions between humans and the fungus exhibit clear divisions. The *C. neoformans* complex is associated with organic components found in the excrement of pigeons, captive birds, dust, and decaying trees of various species. Conversely, exposure to Eucalyptus trees has been the only known ecological niche of *C. gattii* [1,2].

Over the years, the geographic distribution of *C. gattii* complex has widened, leading to its isolation in diverse regions like North America, South America, Asia, and Africa. This expansion of its range can be attributed to factors such as climate change, international travel, and the establishment of the fungus in new ecological niches [2,3].

Historically *C. gattii* has been linked to infections in individuals with a competent immune system. In contrast, *C. neoformans* is often associated with patients affected by HIV/AIDS. Nevertheless, in recent studies, *C. gattii* has been described as equally impacting HIV and non-HIV patients. New risk factors for infection have been identified in these settings, including human immunodeficiency virus HIV, cancer, patients receiving immunosuppressive therapy such as allogeneic transplant receptors, and chronic non-infectious diseases like diabetes mellitus, alcoholism, and smoking [2,4].

Infection in humans occurs through the inhalation of spores or desiccated yeast cells. Once reaching the alveoli, they proceed to accumulate and establish colonies within the respiratory tract, where they may remain in a dormant state for years [5,6]. Within the lung, the fungus undergoes dimorphic changes, allowing it to evade the immune system. Subsequently, it spreads from the primary pulmonary site, disseminating to other organ systems like the skin and central nervous system following a hematogenous route [7].

The term disseminated cryptococcosis applies when the yeast is isolated from more than one organ or system. Clinical manifestation varies based on the site of infection and the host's immune response against the pathogen*.* Systemic features such as fever and weight loss are commonly reported. Neurological involvement commonly manifests as headaches, neck stiffness, and vomiting. Pulmonary infection typically gives rise to symptoms like cough, dyspnea, chest pain, and hemoptysis. Skin involvement often is related to disseminated disease, which is more common in immunocompromised patients. The lesions tend to affect the neck and face [2].

## 2 Case presentation

A 52-year-old previously healthy man sought care at a dermatology office in southeastern Brazil, displaying numerous painless tumors on his face and scalp. Thirty days prior to his visit, he experienced morning headaches, vertigo, fever, cough, and had experienced recent-onset hemoptysis, alongside a substantial 18% reduction in his total body weight. Initially the skin lesions emerged as small papules, which progressively increased in number and size over the course of four weeks, leading to the development of nodules. Initial treatment with cephalosporins proved ineffective.

**Fig. 1:**
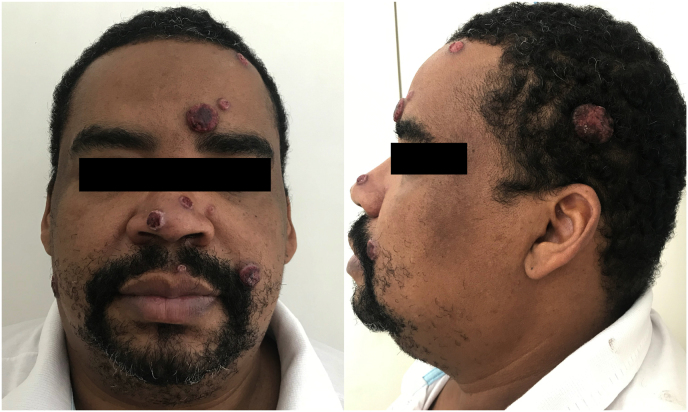
Clinical Image. Erythematous, sessile, dome-shaped nodules covered by crusts distributed on the forehead, cheeks, nose, upper lip, and scalp.

The skin examination unveiled 13 erythematous, sessile, dome-shaped nodules, each covered by crusts distributed across the forehead, cheeks, nose, upper lip, and scalp regions (Fig. 1). Additionally, the neurological assessment revealed a positive Romberg sign, choreoathetosis, photophobia, and double vision. Subsequently, the patient was admitted to the hospital for further investigation (day 0).

**Fig. 2:**
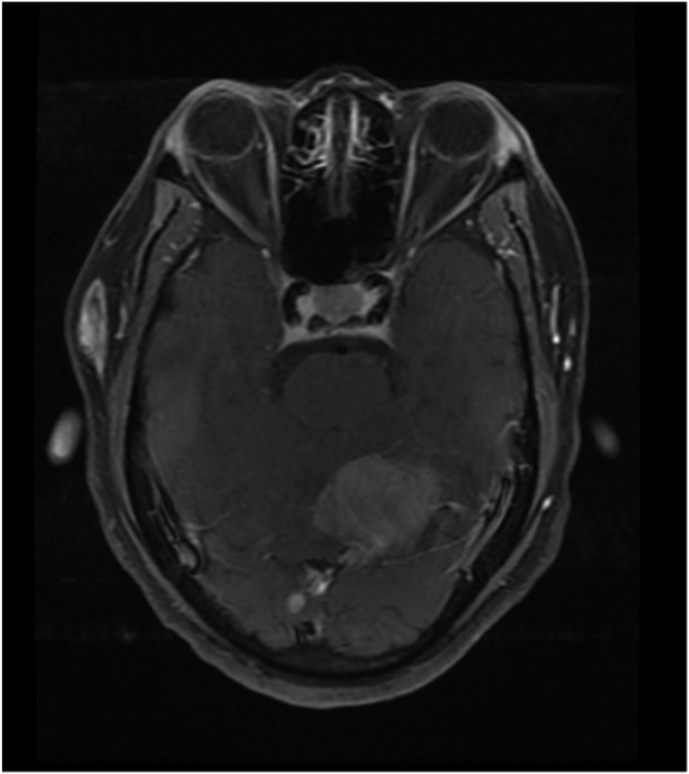
Brain (head) magnetic resonance imaging (MRI). Multiple intra-axial expansive lesions diffused through the supra and infratentorial compartments, with emphasis on lesions in the nucleus capsular region, mesencephalic transition, and in the periphery of the right cerebellar hemisphere, determining an expansive effect with a deviation of the contralateral cerebellar vermis.

**Fig. 3:**
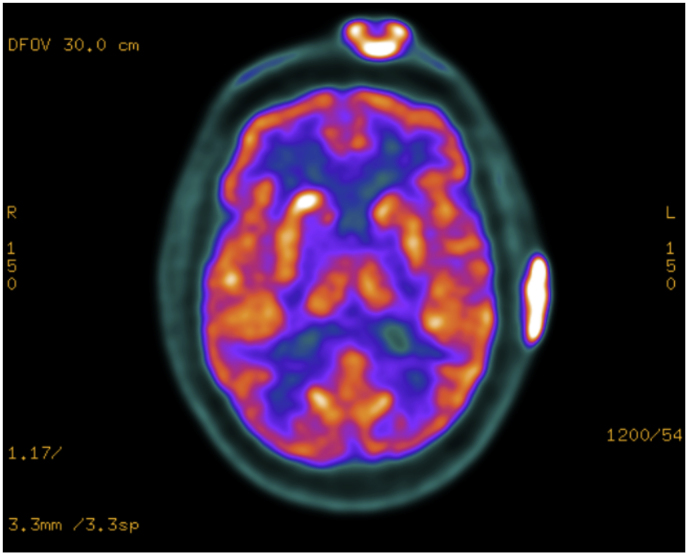
Positron emission tomography (PET) scan Image. Increased glycolytic metabolism in expansive solid lesions with central calcification disseminated in the brain parenchyma, forehead, and scalp.

On the second day of hospitalization, the patient exhibited signs of odynophagia and hoarseness. A fiberoptic nasopharyngoscopy uncovered right vocal fold paramedian paralysis. Further investigation, including brain magnetic resonance imaging (MRI) displayed multiple infra and supratentorial lesions (Fig. 2). Given the potential risk for cerebral herniation, lumbar puncture was avoided. To rule out central nervous system lymphoma, the patient underwent a positron emission tomography (PET) scan, revealing multiple lesions with elevated fluorodeoxyglucose (FDG) uptake in the skin, brain, lung, and vocal cords (Fig. 3). Additionally, a computed tomography (CT) scan highlighted a peripheral lung mass measuring 32 × 40 × 42 mm within the posterior segment of the right lower lobe. A CT-guided percutaneous core biopsy was performed to access this lung mass.

**Fig. 4:**
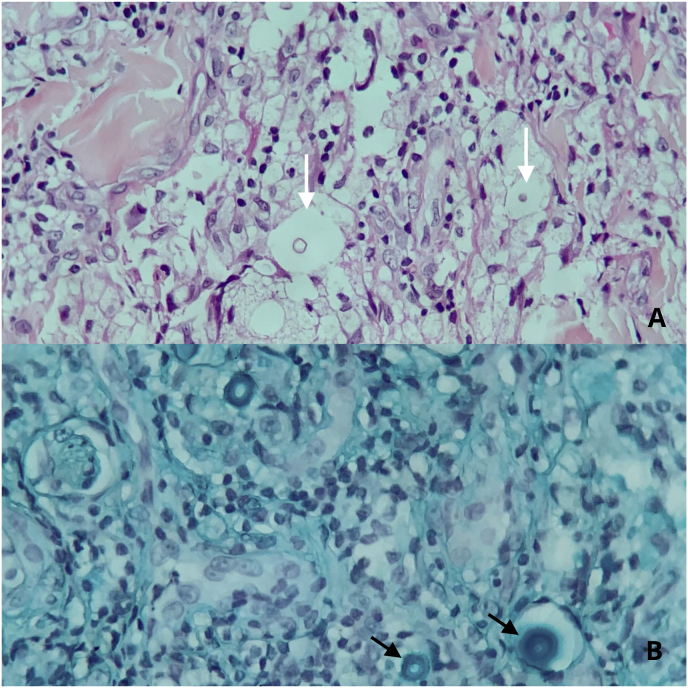
Skin Biopsy **A** – A biopsy specimen of the skin obtained from the patient’s forehead showed numerous yeastlike organisms (white arrows) surrounded by a thick clear mucoid capsule (halo) not stained by H & E − (Hematoxylin and eosin stain. Original magnifications: A–D, × 400). **B** – Highlighting the mucin presence in the *Cryptococcus* capsules (black arrows) – (Alcian blue stain. Original magnifications: A–D, × 400). (For interpretation of the references to color in this figure legend, the reader is referred to the Web version of this article.)

A skin biopsy yielded findings of numerous yeastlike organisms surrounded by a thick clear mucoid capsule – a halo that remained unstained by *hematoxylin and eosin* (Fig. 4). After 72 hours, the mycological culture of both the skin and lung biopsy returned positive, revealing *Cryptococcus.* Additionally, the latex agglutination test and multi-locus sequence typing provided positive results for *Cryptococcus neoformans* var. *gattii* in the blood culture. Confirmation of disseminated cryptococcosis was attained as *C. gattii.* was identified in the skin, blood, and lung samples.

Biochemistry results found normal hemoglobin, white cell count, and renal and liver function tests. Two 4th generation HIV ELISA tests returned negative, and the CD4/CD8 ratio was determined to be 2.12. Clonality research for T cells in both the skin and peripheral blood did not reveal any monoclonal populations. Sputum assessments for acid-fast bacilli and TB gene Xpert testing were negative.

Therapeutic interventions were initiated, including liposomal amphotericin B at a dosage of 3 mg/kg/day IV, 5-fluorocytosine (5-FC) at 25 mg/kg orally four times a day, and dexamethasone at 1mg/kg/day for a four-week induction phase. Encouragingly, the patient’s condition had a favorable outcome, with improved general condition, skin lesions, and neurological symptoms. However, he progressed with secondary nephrotoxicity, characterized by a creatinine clearance of 33.14 mL/min, necessitating aggressive IV fluid rehydration. Moderate anemia with a hemoglobin level of 9.6 g/dL requiring oral treatment involving folic acid and ferrous sulfate tablets.

Following hospital discharge on day 47, a follow-up brain MRI revealed no change compared to previous lesions. Determination of fluconazole’s minimum inhibitory concentrations (MIC) yielded a value of 28 μg/mL. Subsequently, the patient completed the treatment on an oral regimen consisting of voriconazole and 5-FC for 12 months. Follow-up visits were maintained and voriconazole's therapeutic range was kept within 2–4 μg/ml, and the patient tolerated it well, with no evidence of long-term adverse effects.

## 3 Discussion

The skin manifestations of cryptococcosis exhibit a wide range of variations. Among immunocompetent patients, skin lesions can bear a resemblance to molluscum contagiosum, manifesting as umbilicated flesh-colored papules, nodular, acneiform, herpetiform patterns, or keloid-like lesions [6]. The clinical manifestation of the disseminated cryptococcosis in non-HIV-infected patients is likely more closely linked to the host's overall health and immunological status rather than the virulence and pathogenicity of *Cryptococcus* species [8].

Diagnosing cutaneous cryptococcosis can be challenging due to its resemblance to other skin conditions stemming from infectious and non-infectious etiologies. The differential diagnosis must include mycobacterial infections, coccidioidomycosis, blastomycosis, histoplasmosis, basal cell carcinoma, leishmaniasis, Kaposi’s sarcoma, molluscum contagious, and lymphoma. The lesions can involve any part of the body, especially the face and scalp, as seen in this case. Histopathological analysis of skin biopsies with specialized fungal stains, such as periodic acid-Schiff (PAS), Grocott-Gomori methenamine silver (GMS), and Alcian blue stain, is required to discern the presence of yeasts. Mycological cultures and molecular techniques, such as multi-locus sequence typing amplified by polymerase chain reaction (PCR) can aid in confirming the presence of *C. neoformans* var. *gattii* [9].

Patients who present with hoarseness accompanied by dysphagia, odynophagia, hemoptysis, or stridor are considered as having “red flags” and warrant further investigation. The assessment of mass lesions, inflammation, or vocal cord mobility issues, like in our patient, can be achieved by means of visualizing the larynx during a fiberoptic nasopharyngoscopy [10].

Antifungal pharmacotherapy commences with the induction phase, involving amphotericin B and 5-FC, and typically has a duration of 4–6 weeks, depending on the immunocompromised state, antifungal response, and the presence of CNS involvement. Common complications are kidney toxicity, anemia, hypokalemia, and hypomagnesemia. Subsequently, the outpatient consolidation phase entails higher fluconazole dosages or alternative agents, such as voriconazole or posaconazole, extending over a period of 12–18 months [11].

The antifungal susceptibility testing of *Cryptococcus* isolates is not a standard recommendation, given the limited clinical significance of antifungal resistance. However, in cases marked by persistent signs/symptoms or relapses, determination of antifungal resistance is advised. The established breakpoints for fluconazole susceptibility in *Cryptococcus* have (MIC) of ≤8 μg/mL for susceptible species, MIC 16–32 μg/mL for dose-dependent susceptible, and MIC ≥64 μg/mL for resistant isolates [12].

This report highlights the unconventional manifestations displayed by a healthy, immunocompetent individual who exhibited verrucous skin lesions and neurological manifestations due to disseminated cryptococcosis caused by *Cryptococcus neoformans* var. *gattii.* Recognizing and addressing the condition in immunocompetent hosts hinges on timely hospital admission, accurate diagnosis, and prompt initiation of antifungal therapy. Those crucial factors hold the key to avoiding long-term complications and mortality.

## Conflict of interest

There are none.

## References

[1] Siqueira N.P., Favalessa O.C., Maruyama F.H., Dutra V., Nakazato L., Hagen F. (2022 Feb). Domestic birds as source of Cryptococcus deuterogattii (AFLP6/VGII): potential risk for cryptococcosis. Mycopathologia.

[2] Chen S.C., Slavin M.A., Heath C.H., Playford E.G., Byth K., Marriott D. (2012 Sep). Australia and New Zealand Mycoses Interest Group (ANZMIG)-Cryptococcus Study. Clinical manifestations of Cryptococcus gattii infection: determinants of neurological sequelae and death. Clin. Infect. Dis..

[3] Nnadi N.E., Carter D.A. (2021 Apr 29). Climate change and the emergence of fungal pathogens. PLoS Pathog..

[4] Kwon-Chung K.J., Fraser J.A., Doering T.L., Wang Z., Janbon G., Idnurm A. (2014 Jul 1). Cryptococcus neoformans and Cryptococcus gattii, the etiologic agents of cryptococcosis. Cold Spring Harb Perspect. Med..

[5] Noguchi H., Matsumoto T., Kimura U., Hiruma M., Kusuhara M., Ihn H. (2019). Cutaneous cryptococcosis. Med. Mycol. J.

[6] Yang Y., Shen Y.N., Zong W.K., Cui P.G. (2016 Mar-Apr). Disseminated cryptococcosis. Indian J. Dermatol. Venereol. Leprol..

[7] Sabiiti W., May R.C. (2012 Nov). Mechanisms of infection by the human fungal pathogen Cryptococcus neoformans. Future Microbiol..

[8] Nascimento E., Barião P.H.G., Kress M.R.V.Z., Vilar F.C., Santana R.C., Gaspar G.G. (2021 Sep 6). Cryptococcosis by Cryptococcus neoformans/cryptococcus gattii species complexes in non-HIV-infected patients in southeastern Brazil. Rev. Soc. Bras. Med. Trop..

[9] Bardhi R., Lawrence K., Bedford L.M., Deirawan H., Moossavi M. (2020 Dec 15). Disseminated cryptococcosis presenting with cutaneous involvement in an immunocompromised patient. Dermatol. Online J..

[10] Trottier A.M., Massoud E., Brown T. (2013 Nov 19). A case of hoarseness and vocal cord immobility. CMAJ (Can. Med. Assoc. J.).

[11] Henao-Martínez A.F., Chastain D.B., Franco-Paredes C. (2018 Aug). Treatment of cryptococcosis in non-HIV immunocompromised patients. Curr. Opin. Infect. Dis..

[12] Mpoza E., Rhein J., Abassi M. (2017 Nov 26). Emerging fluconazole resistance: implications for the management of cryptococcal meningitis. Med. Mycol. Case Rep..

